# The role of pro- and anti-inflammatory responses in silica-induced lung fibrosis

**DOI:** 10.1186/1465-9921-6-112

**Published:** 2005-10-07

**Authors:** Virginie Barbarin, Aurélie Nihoul, Pierre Misson, Mohammed Arras, Monique Delos, Isabelle Leclercq, Dominique Lison, Francois Huaux

**Affiliations:** 1 Industrial Toxicology and Occupational Medicine Unit, Faculty of Medicine, Université catholique de Louvain, Clos Chapelle-aux-champs 30.54, 1200 Brussels, Belgium; 2 Laboratory of Pathology, University Hospital of Mont Godinne, Université catholique de Louvain, Avenue Dr. G. Thérasse 1, 5530 Yvoir, Belgium; 3 Unit of Gastro-enterology, Faculty of Medicine, Université catholique de Louvain, 53–79, Avenue E. Mounier 53,1200 Brussels, Belgium

## Abstract

**Background:**

It has been generally well accepted that chronic inflammation is a necessary component of lung fibrosis but this concept has recently been challenged.

**Methods:**

Using biochemical, histological, immunohistochemistry, and cellular analyses, we compared the lung responses (inflammation and fibrosis) to fibrogenic silica particles (2.5 and 25 mg/g lung) in Sprague-Dawley rats and NMRI mice.

**Results:**

Rats treated with silica particles developed chronic and progressive inflammation accompanied by an overproduction of TNF-α as well as an intense lung fibrosis. Dexamethasone or pioglitazone limited the amplitude of the lung fibrotic reaction to silica in rats, supporting the paradigm that inflammation drives lung fibrosis.

In striking contrast, in mice, silica induced only a limited and transient inflammation without TNF-α overproduction. However, mice developed lung fibrosis of a similar intensity than rats. The fibrotic response in mice was accompanied by a high expression of the anti-inflammatory and fibrotic cytokine IL-10 by silica-activated lung macrophages. In mice, IL-10 was induced only by fibrotic particles and significantly expressed in the lung of silica-sensitive but not silica-resistant strains of mice. Anti-inflammatory treatments did not control lung fibrosis in mice.

**Conclusion:**

These results indicate that, beside chronic lung inflammation, a pronounced anti-inflammatory reaction may also contribute to the extension of silica-induced lung fibrosis and represents an alternative pathway leading to lung fibrosis.

## Background

Lung fibrosis is often associated with an inflammatory process which precedes or coexists with fibroblast proliferation and deposition of extracellular matrix proteins [1]. Abundant human and experimental data have highlighted inflammation as a major effector in the development of lung fibrosis [2-4]. Persistent inflammation characterized by an accumulation of macrophages, neutrophils and lymphocytes in the lung causes the release of degradative enzymes and oxidants capable of inducing lung injury and DNA damage [5,6]. Lung inflammatory cells are also a source of growth factors [7], cytokines [8] and chemokines [9] that amplify and maintain alveolitis and activate fibroblasts. It has been demonstrated, for instance, that macrophages obtained from animal models of silicosis [10] or from patients with lung fibrosis [11] overproduce pro-inflammatory cytokines and growth factors such as TNF-α, IL-1, PDGF and TGF-β. All these mediators clearly possess strong stimulating activities on fibroblasts [4,12].

Beside this strong evidence of a major role of inflammation, other studies did not find a clear relationship between lung inflammation and fibrosis and, thus, have challenged this paradigm.

First, several studies, mainly conducted in mice, reported that lung inflammation is not always followed by a fibrotic disease. Adamson and colleagues showed that an increased pulmonary inflammation induced by the leukocyte chemoattractant FMLP (N-formyl-L-methionyl-leucyl-phenylalanine), significantly reduced the fibrotic response induced by silica in mice [13]. In addition, IL-10 deficient mice treated with silica showed an intense alveolitis but a reduced fibrotic lung response compared to their wild-type counterparts [14]. Also, αvβ6 integrin knockout mice developed marked lung inflammation in response to bleomycin but failed to develop fibrosis [15].

Second, the control of inflammation is not always associated with a reduction of fibrosis. In mice, treatment with anti-MIF (macrophage migration inhibitory factor) antibodies significantly reduced the accumulation of inflammatory cells in the alveolar space as well as TNF-α production after treatment with bleomycin but did not affect the lung fibrotic response [16]. Also, IL-12p40^-/- ^mice treated with bleomycin exhibited reduced pulmonary inflammation but increased fibrosis compared to the wild type mice [17].

Finally, in humans, anti-inflammatory therapy has never been definitely shown to significantly alter the course of pulmonary fibrosis [18,19].

Collectively, these observations suggest that inflammation is not necessarily related to the fibrotic response and that additional pathogenic routes can be responsible for the development of a pulmonary fibrotic response. Alternative paradigms have therefore been proposed [20,21].

It has been postulated that pulmonary fibrosis may result from sequential epithelial cell injury and abnormal wound repair, independently from an inflammatory reaction [22]. Angiogenesis may also constitute a pivotal process in the development of lung fibrosis, independent of lung inflammation [23]. We have recently proposed a third pathogenic pathway which is based on the profibrotic activity of anti-inflammatory cytokines such as IL-4, IL-13, TGF-β but also IL-10 [24]. Indeed, these cytokines stimulate, directly or indirectly, the fibroblasts to proliferate and/or to produce extracellular matrix proteins. Their protracted overproduction in the lung, e.g. in response to a sustained insult, may in turn drive a fibrotic process.

In this study, we compared the lung responses to silica in Sprague-Dawley rats and NMRI mice, two animal models largely used to study the pathogenesis of lung fibrosis [25,26]. By comparing these species, we show that the extension (rats) but also the control of the inflammatory reaction (mice) induced by silica particles may both lead to the emergence of similar fibrotic lesions.

## Methods

### Animals

Female NMRI mice and Sprague-Dawley rats weighing respectively 20 to 25 g and 200 to 300 g, were purchased from Charles River (Brussels, Belgium). Female BALB/c, C57BL/6J, and DBA2 mice were obtained from our local breeding facility (Ludwig Institute, Brussels). The animals were housed in positive pressure air-conditioned units (25°C, 50% relative humidity) on a 12-hr light/dark cycle. The experimental protocol was approved by the local committee for animal use at the Université catholique de Louvain.

### Instillation method

To allow sterilization and inactivation of any trace of endotoxin, particles were heated at 200°C for 2 h immediately before suspension and administration.

A suspension of crystalline silica particles (DQ12; d50 = 2.2 μm, a gift from Dr L. Armbruster Essen, Germany) in sterile 0.9% saline was injected directly into the lungs of mice and rats by intratracheal instillation. To allow a comparison between both species, the doses of silica were adjusted to administer 2.5 and 25 mg of silica per g of lung (lung weight; mouse about 200 mg and rat about 1.2 g; ratio = 6; instillation of 0.5 or 5 mg silica in 60 μl of saline in mice and 3 or 30 mg silica in 360 μl saline in rats). These doses of silica are comparable to those usually used in the literature to induce intense lung fibrosis [27,28]. All instillations were performed on anesthetized animals after surgical opening of the neck. Two mg of sodium pentobarbital (Certa, Braine-l'Alleud, Belgium) or a mix of 10 mg of Ketalar (N.V. Warner-Lambert, Zaventem, Belgium) and 2 mg of Rompun (Bayer A6, Leverkussen, Germany) were used to anesthetize rats or mice, respectively.

For the particle comparative model, 2.5 mg of silica, tungsten carbide (WC, d50 = 1 μm) or manganese dioxide (MnO_2_, d50 = 3.7 μm) particles in sterile 0.9% saline (60 μl) were injected directly into the lungs of mice by intratracheal instillation.

### Bronchoalveolar lavage and whole lung homogenates

At selected time intervals after silica treatment (3, 30 and 60 days), mice and rats were sacrificed with sodium pentobarbital (20 mg/mice and 120 mg/rat, i.p) and a bronchoalveolar lavage (BAL) was performed by cannulating the trachea and infusing the lungs twice with sterile 0.9 % saline. The volume of saline used for BAL was determined on the basis of the lung weight (ratio between rat and mouse weight = 6). Since mice are usually lavaged with 1 ml, we used 6 ml for lavaging the rats. BAL fluid fractions were centrifuged (1500 rpm, 10 min, 4°C) and the cell-free supernatant of the first fraction was used for biochemical measurements. The cell pellets of the two BALF fractions were pooled and resuspended with 2 ml of sterile saline for mice and 12 ml for rats. Aliquots of the cell suspensions were used to determine cell numbers (200 cells counted). Cell differentials were performed on cytocentrifuge preparations fixed in methanol and stained with Diff-Quik (Baxter, Lessines, Belgium).

Separately, at day 3, 30 or 60 after treatment, non-lavaged whole lungs were perfused and excised. The right lobes were placed into a Falcon tube chilled on ice and 3 ml (mice) or 18 ml (rats) of cold 0.9% NaCl were added. The content of each tube was then homogenized with a Ultra-Turrax T25 homogenizer (Janke and Kunkel, Brussels, Belgium) during 30 second. The homogenates were kept frozen at -80°C until use.

The time points analyzed in this study were selected to correspond to the peaks of inflammation (3 days) and fibrosis (2 months) in the mouse and rat models [25,29,28].

### Biochemical analyses

Lactate dehydrogenase (LDH) activity in BALF was assayed spectrophotometrically by monitoring the reduction of nicotinamide adenine dinucleotide (NAD^+^) at 340 nm in the presence of lactate. Total proteins in BALF were determined by the pyrogallol red staining method (Technicon RA system; Bayer Diagnostics, Domont, France).

### Silica measurement

The amount of silica particles remaining in the lungs of rats and mice was measured after 3, 30 and 60 days following administration. The concentration of silica was determined colorimetrically with the molybdenum blue method after digestion in sodium hypochlorite [30].

### Anti-inflammatory therapy

Dexamethasone (2.5 μg/ml) was administered in the drinking water starting 3 days before silica (25 mg/ g lung) or saline instillation both in rats (0.25 mg/kg/d) and mice (0.375 mg/kg/d). Two times per week throughout the experimental protocol, 1.25 mg of dexamethasone phosphate (Sigma) was diluted to 500 ml of drinking water [31]. Pioglitazone (Takeda, Japan, commercialized by Eli Lilly, Belgium) was added to powdered standard rodent chow (0.01% wt/wt, ad libitum) [32]. This treatment started 3 days before silica or saline pulmonary administration (10 and 15 mg/kg/d in rats and mice, respectively). Control animals were given powdered standard lab chow ad libitum and tap water. The selection of dose of anti-inflammatory molecules were based on those reported in the literature to significantly attenuate inflammation in rats and mice [31,33,34,32]. Two months after silica or saline treatment, animals were sacrificed and BALF inflammatory parameters (see above) as well as lung collagen deposition (see below) were quantified.

### Collagen assay

Collagen deposition was estimated by measuring the lung hydroxyproline content. Lung homogenates were hydrolyzed in 6N HCl overnight at 110°C. Hydroxyproline was assessed by high- performance liquid chromatography analysis [35] and data are expressed as micrograms of hydroxyproline per ml of lung homogenate.

### Enzyme-linked immunosorbent assays (ELISA)

Type I collagen contents were measured in lung homogenate supernatants (5000 rpm, 4°C, for 10 min) using standardized ELISA as previously described [36].

Mouse and rat IL-10 (Biosource International, Camarillo, CA, USA), TNF-α (Pharmingen, BD Biosciences, San Diego, USA) concentrations were measured in lung and BAL supernatants using ELISA kits following the manufacturer's protocols. The detection limits of these ELISA are respectively 0.9, 5, 5 and 5 (pg/ml).

### Histopathology and immunohistochemical staining

The left lung of silica-treated or control mice was excised and fixed in Bouin solution (Merck-Belgolabo, Belgium). Paraffin-embedded sections were stained with hematoxylin and eosin or Masson's trichrome for light microscopic examination. For immunohistochemistry stainings, dewaxed and rehydrated tissue sections were subjected to endogenous peroxidase inactivation (0.5% H_2_O_2 _for 20 min) followed by three washes of 5 minutes in PGT buffer (phosphate-buffered saline [PBS], 0.05% Tween 20, and 0.02% gelatine). An incubation was then performed for 1 h in a humidified room with a rat monoclonal anti-mouse IL-10 antibody (SXC1) diluted 250 times in PBS. After 3 washes with PGT buffer (5 min each), tissue sections were exposed for 1 h to the second antibody (polyclonal rabbit against rat IgG coupled with peroxidase as second antibody, Dako, Copenhagen, Denmark) diluted 40-fold in PBS supplemented with 1% mouse serum. Tissue sections were then rinsed and washed three times in PGT buffer. The peroxidase activity was revealed by 3-3'-diaminobenzidine tetrahydrochloride (Aldrich, Beerse, Belgium)-H_2_O_2 _substrate. The staining was enhanced by incubation in a solution of 0.5% CuSO_4 _in saline for 15 min. Sections were counterstained with Harris hematoxylin, rinsed, dehydrated, and mounted in DPX (BDH, Poole, UK).

### Statistics

Treatment-related differences were evaluated using *t *tests and one-way analysis of variance, followed by pairwise comparisons using the Student-Newman-Keuls test, as appropriate. Statistical significance was considered at *P *< 0.05.

## Results

### Pulmonary inflammation induced by silica particles was persistent in rats but limited in mice

**Figure 1:**
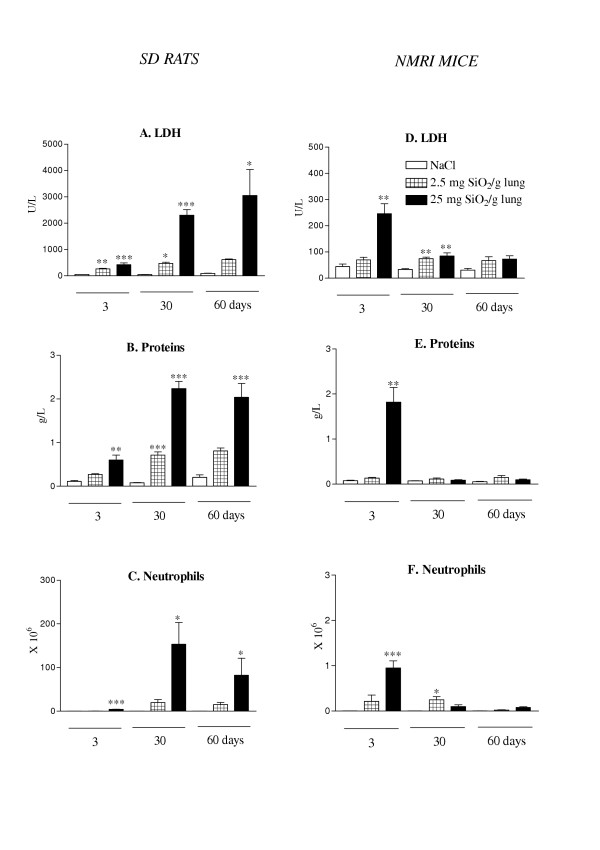
Lactate dehydrogenase (LDH) activity, total protein content and neutrophil numbers in bronchoalveolar lavage of Sprague-Dawley (SD) rats (A-C) and NMRI mice (D-F) after intratracheal instillation of saline or silica particles (2.5 or 25 mg/ g lung). Bars represent means +/- SEM of 5 to 7 animals. Significant differences between treated animals and controls: *P < 0.05, **P < 0.01, ***P < 0.001 (Student-Newman-Keuls multiple comparison test). Please note the different scales between mice and rats for LDH and neutrophils.

LDH activity, protein levels and neutrophil numbers measured in BALF were used to estimate the amplitude of pulmonary inflammation induced by silica particles both in rats and mice (2.5 and 25 mg/g of lung) (Figure 1). At all time points and in a dose-dependent manner, silica induced a significant increase in BALF LDH and protein levels in rats (Figure 1 A &1 B). Similarly, an accumulation of lung neutrophils (Figure 1 C), macrophages and lymphocytes (data not shown) was observed in silica-treated rats in a dose-related manner. These effects were progressive and the most pronounced 30 and 60 days after particle treatment. Thus, these observations showed the establishment of a chronic alveolitis in silica-treated rats.

In mice, BALF LDH and protein levels were found increased only at day 3 after silica treatment and both parameters had returned to control values after 60 days. A dose-dependent recruitment of pulmonary neutrophils was noted at day 3 in the BALF of silica-treated mice but this neutrophil accumulation did not last after 30 days. The modifications of BALF numbers of macrophages and lymphocytes were similar to that observed with neutrophils (data not shown). On the basis of these results, we concluded that mice controlled silica-induced alveolitis and did not develop chronic inflammation.

### Rats and mice developed a similar lung fibrotic reaction to silica

**Figure 2:**
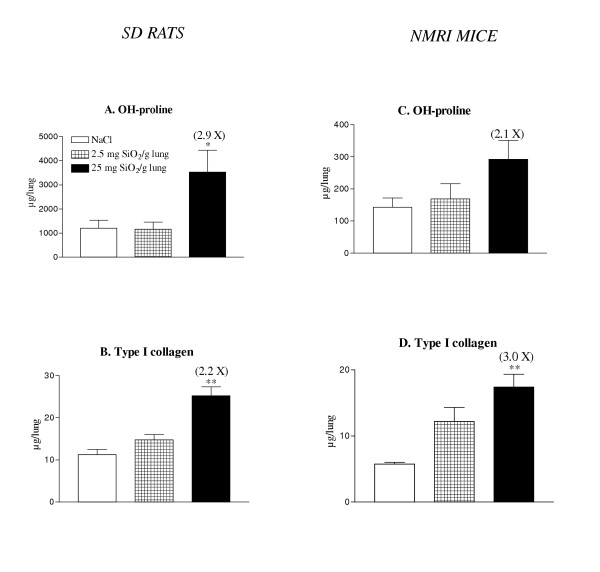
Hydroxyproline and type 1 collagen contents in lung homogenates of SD rats (A-B) and NMRI mice (C-D) 60 days after intratracheal instillation of saline or silica particles (2.5 or 25 mg/ g lung). Bars represent means +/- SEM of 5 to 7 animals. Significant differences between treated animals and controls: *P < 0.05, **P < 0.01, ***P < 0.001 (Student-Newman-Keuls multiple comparison test).

**Figure 3:**
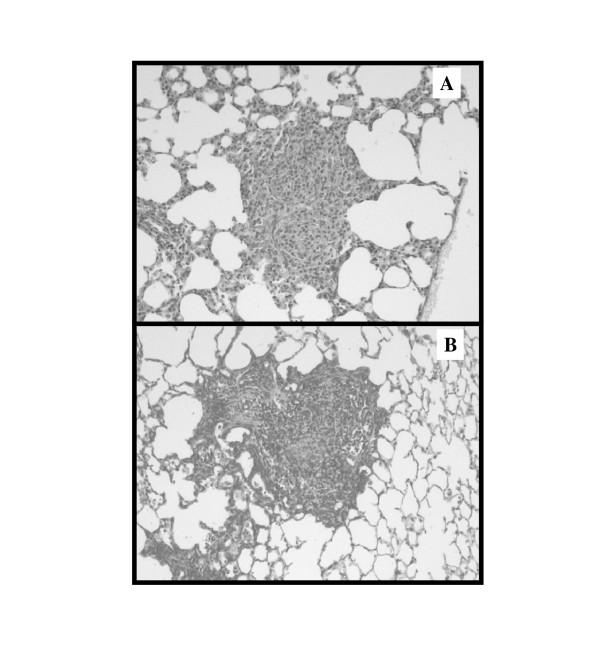
Representative silicotic nodules from (A) SD rats and (B) NMRI mice 60 days after silica instillation (25 mg/g lung). Masson trichrome staining. Magnification 200X.

To estimate silica-induced lung fibrosis, hydroxyproline and type-1 collagen levels were measured in lung homogenates in both species. A clear accumulation of extracellular matrix components was noted both in rats and mice after 60 days (Figure 2). The amplitude of the lung fibrotic reaction was relatively similar in both species since, at the highest dose tested, silica induced a 2.9- and 2.1-fold increase in OH-proline, in rats and mice respectively (Figure 2 A &2 C). Type-1 collagen contents were 2.2- and 3-fold increased in silica-treated rats and mice, respectively (Figure 2 B &2 D). As shown in Figure 3, both species developed clear silicotic lesions characterized by the formation of well defined and organized silicotic nodules. No significant lung fibrosis was noted at days 3 and 30 in the two silica-treated species (data not shown).

These results indicated therefore that, while rats and mice treated with equivalent doses of silica developed contrasting inflammatory responses, both species showed in turn similar lung fibrotic reactions.

### The lung persistence of silica particles was similar in rats and mice

The amplitude of silica-induced lung inflammation and fibrosis directly depends on the amount of particles retained in the lung. We therefore assessed the amount of silica particles that remained in the lungs in both species. Similar amounts of silica particles were retrieved 30 and 60 days after treatment in both rats and mice (day 30, respectively 96.9 ± 9.8 and 80.4 ± 24.9 percent of the mean silica content measured 3 days, values represent means ± SEM; day 60, respectively 113.1 ± 22.6 and 110.3 ± 28.5 %; n = 5 to 7). On the basis of these data, we excluded a difference in the clearance of silica particles to explain the varying lung responses between rats and mice.

### The lung response to silica was characterized by the production of pro-inflammatory mediators (TNF-α) in rats and of anti-inflammatory mediators (IL-10) in mice

**Figure 4:**
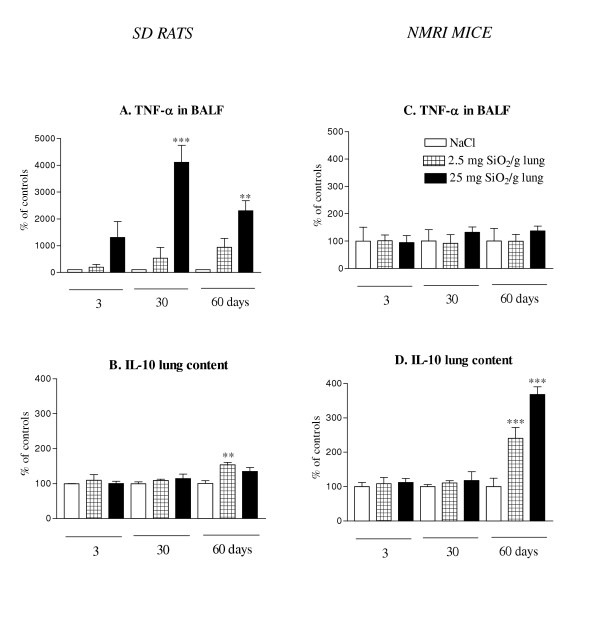
Levels of TNF-α in BALF and IL-10 in lung homogenates of SD rats (A-B) and NMRI mice (C-D) after intratracheal instillation of saline or silica particles (2.5 or 25 mg/ g lung). Bars represent means +/- SEM of 5 to 7 animals. Significant differences between treated animals and controls: **P < 0.01, ***P < 0.001 (Student-Newman-Keuls multiple comparison test). At day 3, absolute levels of TNF-α in saline rats and mice were respectively 6.3 ± 0.7 and 11.7 ± 1.2 pg/ml. For IL10, 175.5 ± 1.2 and 3618.1 ± 422.8 pg/ml were respectively detected in saline rats and mice (day 3).

It is well demonstrated that pro-inflammatory cytokines such as TNF-α are involved in the pathogenesis of silica-induced lung disease [12]. TNF-α levels were therefore measured in BALF and lung homogenates of rats and mice treated with silica (Figure 4 A–C). A dose-dependent and progressive increase of TNF-α in BALF was observed after silica treatment in rats (Figure 4 A). Similar data were obtained by measuring TNF-α in lung homogenates by ELISA or by assessing the amounts of TNF-α transcripts (semi-quantitative RT-PCR) in BALF cells and whole lungs (data not shown). In striking contrast, no such induction was found in BALF (Figure 4 C) or lung homogenates (data not shown) of silica-treated mice. Since it is well demonstrated that the anti-inflammatory cytokine IL-10 may downregulate the expression of TNF-α, we assessed IL-10 levels in the BALF and lung homogenates of silica-treated rats and mice (Figure 4 B–D). A significant and dose-dependent increase of IL-10 production was observed in lung homogenates of silica-treated mice at day 60 (Figure 4 D). No similar IL-10 induction was noted in treated rats (Figure 4 B). Moreover, it was noteworthy that the basal IL-10 content (saline) was 20-fold higher in the lung of mice than in rats (at day 3, saline rats: 175.5 ± 1.2 vs saline mice: 3618.1 ± 422.8 pg/ml). IL-10 was not detected in BALF neither in rats nor in mice. The differences in IL-10 expression were confirmed by semi-quantitative RT-PCR in BALF cells and lung homogenates (data not shown). No similar effect on the levels of IL-4, IL-13 or IFN-γ was noted in this model. Altogether, these results indicated that the mediators associated to the lung response to silica were opposite in both species. The rat lung response was characterized by the expression of a pro-inflammatory cytokine such as TNF-α while the mouse lung response involved an anti-inflammatory cytokine such as IL-10. These observations also suggested that the limited lung inflammation observed in mice could be related, at least in part, to their increased expression of IL-10.

### IL-10 expression was intimately related to silica-induced lung fibrosis in mice

**Figure 5:**
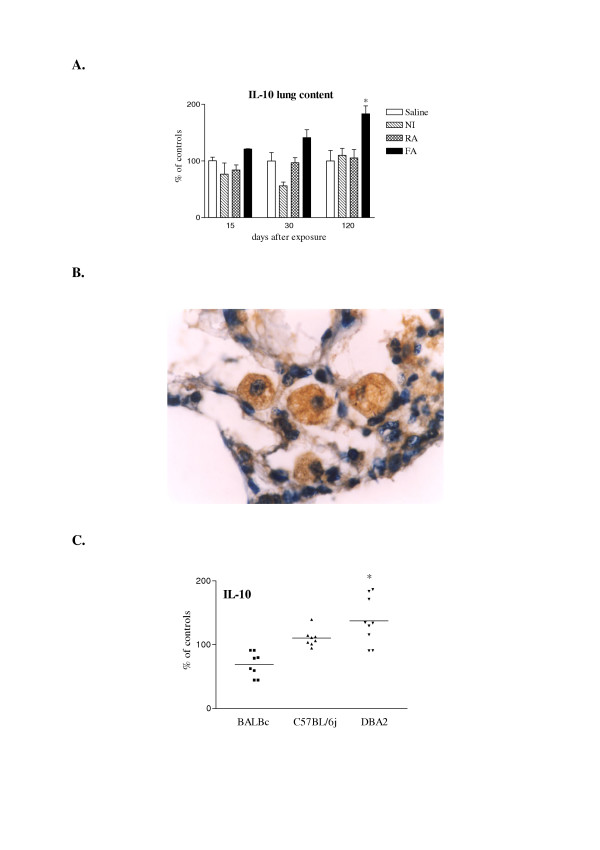
**A**: Time-dependent IL-10 production in the lung of NMRI mice in the fibrosing alveolitis (FA), resolving alveolitis (RA) and non-inflammatory models (NI). Bars represent means +/- SEM of 5 to 6 animals. Significant differences from controls: *P < 0.05 (Student-Newman-Keuls multiple comparison test). **B**: Cellular immuno-localization of IL-10 production in the FA model at day 120. Mainly macrophages having phagocytozed silica particles expressed IL-10. **C**: IL-10 contents in lung homogenates of BALBc, C57BL/6 and DBA/2 mice 60 days after intratracheal instillation of silica (2.5 mg). DBA2 mice developed the most severe fibrotic lesions and expressed the highest IL-10 levels. The results for individual mice are shown. The bars denote the mean values for each group (n = 8 to 9). Significantly different from controls: *P < 0.05 (Student-Newman-Keuls multiple comparison test).

In order to further explore the role of IL-10 in the establishment of lung fibrosis in mice, we used a mouse model that allows a comparison among three different types of particles (tungsten carbide, WC; manganese dioxide, MnO_2_; and crystalline silica, SiO_2_) and the identification of specific events leading to the extension of lung fibrosis [37]. After intratracheal instillation of these mineral dusts, the pulmonary responses in NMRI mice were characterized respectively by no inflammation (NI), resolutive alveolitis (RA) or fibrosing alveolitis (FA). As already observed, a persisting increase of IL-10 production was observed in the FA model (silica) which paralleled the establishment of lung fibrosis (Figure 5 A). No significant change in lung IL-10 content was noted in the saline, NI (poorly soluble particles of low toxicity, WC) or RA (inflammatory but not fibrogenic particles) groups, indicating that IL-10 induction in mice seems specific to the fibrotic process. No similar effect on the levels of IL-4, IL-13 or IFN-γ was noted in this comparative model.

To determine the localization and cellular sources of IL-10 in the fibrotic model (FA), we evaluated lung tissue sections obtained at the late stage of the disease (120 days). This analysis showed that, in silica-treated mice, alveolar macrophages appeared as the major cells expressing IL-10. The corresponding sections showed no staining with control non-immune IgG (data not shown). Using polarized light and high magnification, we found that mainly macrophages that had phagocytozed silica particles expressed IL-10 (Figure 5 B).

To complete the data obtained in mice, we also determined the IL-10 levels in the lung of several strains of mice presenting different sensitivities to silica [38]. BALB/c, C57BL/6J and DBA2 mice received silica particles (2.5 mg/mouse) or saline. Two months after treatment, collagen deposition and histology were assessed to monitor the lung fibrotic response. As previously described [38], we observed that DBA2 mice developed the most severe fibrotic lesions. While C57BL/6 mice developed intermediate lung fibrosis, BALB/c mice responded weakly to silica. This gradient of susceptibility was illustrated by measuring pulmonary collagen contents and by histological analysis (data not shown). Lung IL-10 levels were related to the amplitude of pulmonary fibrosis. Indeed, IL-10 contents were significantly higher after silica treatment in lung homogenates of DBA2 mice in comparison to their respective controls (Figure 5 C). While no difference between saline- and silica-treated groups was noted in C57BL/6, in BALB/c mice silica treatment induced a reduction of IL-10 contents in comparison to saline (Figure 5 C). Levels of IL-10 in lung tissue of saline mice were as follows: BALB/c = 605 ± 36; C57BL/6 = 683 ± 62 and DBA/2 = 1049 ± 123 pg/lung. No similar effect on the levels of IL-4, IL-13 or IFN-γ was noted in this comparative model.

Altogether, we concluded that expression of IL-10 was intimately correlated with the amplitude of silica-induced lung fibrosis.

### Reduction of inflammation prevented silica-induced lung fibrosis in rats but not in mice

**Figure 6:**
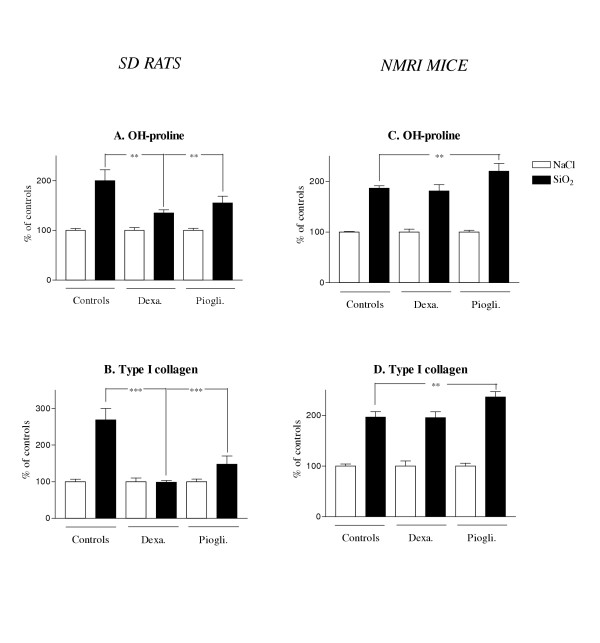
Hydroxyproline and type 1 collagen contents in lung homogenates of SD rats (A-B) and NMRI mice (C-D) 60 days after intratracheal instillation of saline or silica particles (2.5 or 25 mg/ g lung) and after dexamethasone or pioglitazone treatment. Bars represent means +/- SEM of 5 to 7 animals. Significant differences between treated animals and controls: *P < 0.05, **P < 0.01, ***P < 0.001 (Student-Newman-Keuls multiple comparison test).

Our observations suggested two opposite lung responses in association with the development of silica-induced lung fibrosis (inflammatory and anti-inflammatory, respectively in rats and mice). In order to delineate the role of lung inflammation in both models of lung fibrosis, we treated silica-administered rats and mice with anti-inflammatory molecules. Dexamethasone (corticosteroids) and pioglitazone (a peroxisome proliferator-activated receptor-gamma agonist) were used in this study because they have been shown to control lung inflammation [33,34]. In rats, silica-induced lung fibrosis was significantly reduced both after dexamethasone or pioglitazone treatment as estimated by OH-proline or type I collagen lung levels (Figure 6 A and 6 B). This reduction of lung fibrosis was accompanied by a limited accumulation of leukocytes in the lung but not by an amelioration of biochemical parameters (i.e., LDH and protein BALF levels; at day 60) (data not shown). In striking contrast, the anti-inflammatory treatments had no similar effect on the amplitude of lung fibrosis in silica-treated mice. Moreover, pioglitazone administration increased the pulmonary levels of OH-proline and type I collagen after silica (Figure 6 C and 6 D), denoting an exacerbated lung fibrotic process in this group. No significant effect of the anti-inflammatory treatments was observed on lung inflammatory parameters as well as cytokine production (IL-10, IL-4, IL-13 and IFN-γ) in mice. Altogether, these data indicated that the inflammatory process drives the pathogenesis of lung fibrosis in rats but not in mice.

## Discussion

Lung fibrosis, in humans as well as in experimental models, is often associated with pulmonary inflammation characterized by the accumulation of macrophages, lymphocytes and granulocytes [1]. These inflammatory cells release toxic oxygen derivatives and proteolytic enzymes which cause cellular damage and disruption of the extracellular matrix which, in turn, leads to destroying lung architecture [39]. Inflammatory cells are also considered to be a major source of mediators such as cytokines and growth factors, which possess the ability to stimulate fibroblast functions critical to fibrogenesis [1].

This scenario was largely described in models of lung fibrosis induced by silica or asbestos mainly in rats [40]. Indeed, compelling evidence demonstrates that pro-inflammatory cytokines such as IL-1 and TNF-α not only regulate chronic lung inflammation but also fibrosis. For instance, activated alveolar macrophages purified from rats instilled with silica particles release pro-inflammatory mediators such as IL-1 and TNF-α as well as MIP-2, responsible for the persistence of inflammation and the development of fibrosis [10,41]. The fact that lung inflammation and production of pro-inflammatory cytokines leads to the development of fibrosis was also reported in models using bleomycin or ionizing radiation to induce lung fibrosis [12,42]. In the present study, we also found that Sprague-Dawley rats injected with silica particles developed progressive inflammation, with a dramatic accumulation of neutrophils at the latter stage of the disease. As already demonstrated [10,29], the lung response to silica in rats was accompanied by an overproduction of TNF-α. The key role of the inflammatory reaction in the extension of lung fibrosis induced by silica was demonstrated by the efficacy of anti-inflammatory therapy in this study. Indeed, in rats, silica-induced fibrosis was strongly attenuated by dexamethasone or pioglitazone administration (Figure 6).

By contrast, in the mouse model used in this study, the lung response to silica was not associated with chronic inflammation or a significant up-regulation of TNF-α expression. This control of inflammation in mice was accompanied by a pronounced expression of IL-10, both at the basal level as well as in response to silica. The fact that this marked overproduction of IL-10 in the lung may contribute to limit inflammation induced by silica is largely supported by existing experimental studies. In mice genetically deficient in IL-10, we have previously reported that the administration of silica particles induced an enhanced inflammatory reaction compared to wild type animals [14]. Furthermore, IL-10 has been shown to suppress tissue inflammation in other mouse models of lung insult induced by Pneumocystis Carinii infection [43], endotoxin [44], bleomycin [45] or immune complexes [46]. The absence of response to anti-inflammatory drugs in mice further supports the fact that fibrosis was not driven by inflammation in this species.

Our data indicate that despite its anti-inflammatory properties, IL-10 participates to the extension of the fibrotic reaction. An obvious association between the extent of IL-10 overexpression and the amplitude of fibrosis was shown in this study. First, alveolar macrophages, strongly implicated in the pathogenesis of lung fibrosis induced by silica particles [47], were identified as the main cellular source of IL-10 in mice (Figure 5 B). Moreover, using several mineral particles inducing different lung responses, we showed that IL-10 production in the lung of mice was up-regulated during the development of fibrosing alveolitis (FA, silica) but not in the resolutive alveolitis (RA, MnO_2_) or the non-inflammatory models (NI, WC) (Figure 5 A). In addition, IL-10 was up-regulated in silica-susceptible mice (DBA2) but reduced in a resistant strain (BALB/c) (Figure 5 C). It is noteworthy that these last characteristics recapitulate those reported for the pro-fibrotic and pro-inflammatory cytokine TNF-α in the rats. Indeed, TNF-α is overproduced mainly by activated lung macrophages, induced in the rat lung only by fibrotic particles [10], and overexpressed in silica-treated sensitive animals [48,27]. Together with our previous observations demonstrating a pro-fibrotic activity of IL-10 in the lung of mice treated with silica particles [14,24,49], we can conclude that the overexpression of IL-10 documented in the lung of mice in the present study contributed to the establishment of the fibrotic response.

The mechanism by which IL-10 exerts its pro-fibrotic effect is still unclear. The possibility that IL-10 may directly stimulate fibroblasts has already been tested with conflicting results. Thus, Liu and colleagues showed that IL-10 had no significant effect on human fetal lung fibroblasts [50]. In contrast, it has been reported that IL-10 might downregulate type I procollagen mRNA expression in skin fibroblasts [51], as well as constitutive and TGF-β-stimulated type I collagen mRNA expression in a human lung fibroblast cell line [45]. By using primary cultures of mouse lung fibroblasts, we previously showed that unlike other Th2 cytokines such as IL-4 and IL-13 [52,53], IL-10 did not directly modulate fibrosis-associated functions, such as proliferation and collagen or α-SMA expression [24]. IL-10 could, however, exert its pro-fibrotic action by up-modulating the expression of pro-fibrotic mediators such as TGF-β. Indeed, in IL-10 transgenic mice, the expression of TGF-β was found increased in the lung [54], which is consistent with our previous study showing a significant reduction of TGF-β lung levels in IL-10 deficient mice exposed to silica [24]. Moreover, IL-10 enhances the expression of the type II TGF-β receptor and restores TGF-β responsiveness on activated T cells [55] but similar effects have not been explored in fibroblasts. Recent work from this laboratory showed that, in mice treated with silica, lung overexpression of IL-10 upregulated the production of other Th2 cytokines such as IL-4 and IL-13. Since there is evidence that pulmonary fibrosis is a Th2-mediated process, we speculate that elevated lung levels of IL-10 may also contribute to the progression of fibrosis via its capacity to stimulate Th2 polarized responses. Collectively, these observations strongly indicate that IL-10, an anti-inflammatory/Th2 cytokine, exacerbates the severity and pathology of lung fibrosis, at least in the mouse.

## Conclusion

On the basis of the comparative models studied here, we suggest that at least two different types of lung response to silica can lead to the development of lung fibrosis. First, as observed in the Sprague-Dawley rats, lung fibrosis is the consequence of a chronic and exaggerated inflammatory response associated with an overproduction of pro-inflammatory mediators also possessing pro-fibrotic activities such as TNF-α. In mice, marked expression of anti-inflammatory cytokines such as IL-10 have beneficial effects by limiting and controlling inflammation. However, in this species, because of its pro-fibrotic properties, IL-10 participates to the extension of fibrosis. Thus, in mice, the strong anti-inflammatory response established to control inflammation could contribute to the fibrotic reaction induced by silica. These data clearly suggest that several pathogenic routes are responsible for the development of a pulmonary fibrotic response.

It remains however, to learn how to extrapolate these observations to human diseases. Anti-inflammatory therapies used in this study had no effect on lung fibrosis in mice while they were very efficient in the control of the disease in rats. This may indicate that the treatment of pulmonary fibrosis may need to be modulated according to the type of pathogenic mechanism involved.

## Competing interests

The author(s) declare that they have no competing interests.

## Authors' contributions

VB: planned the experimental design and drafted the manuscript.

AN: participated in the study design and performed biochemical and cellular studies.

PM: participated in the study design and performed biochemical and cellular studies.

MA: participated in the study design and performed animal studies.

MD: participated in the study design and performed histological studies.

IL: participated in the study design and performed anti-inflammatory therapy.

DL: participated in the study design, helped to draft the manuscript and coordinated the research group.

FH: participated in the study design, helped to draft the manuscript and coordinated the research group.
